# Efficacy of music therapy on stress and anxiety prior to dental treatment: a systematic review and meta-analysis of randomized clinical trials

**DOI:** 10.3389/fpsyt.2024.1352817

**Published:** 2024-02-23

**Authors:** Nansi López-Valverde, Antonio López-Valverde, Bruno Macedo de Sousa, José Antonio Blanco Rueda

**Affiliations:** ^1^ Department of Surgery, Instituto de Investigación Biomédica de Salamanca (IBSAL), University of Salamanca, Salamanca, Spain; ^2^ Institute for Occlusion and Orofacial Pain Faculty of Medicine, University of Coimbra, Coimbra, Portugal

**Keywords:** dental anxiety, dental fear, dental phobia, stress, music therapy, randomized clinical trial, meta-analysis

## Abstract

**Introduction:**

Stress and anxiety are emotional states that often accompany patients who have to receive dental treatments, leading them to postpone or avoid treatments with the consequent deterioration of their oral health and, hence, their general condition. Music therapy has been shown to be an alternative to other treatments that are invasive and not without danger, such as anxiolytics or sedation. This systematic review and meta-analysis evaluated the effect of music therapy on anxiety and stress prior to dental treatments.

**Methods:**

Studies published in PubMed (through Medline), Web of Science (WOS), Embase, and Cochrane Library databases were consulted up to October 2023. The inclusion criteria were established for intervention studies (randomized controlled trials, RCTs) according to the PICOS (population, intervention, comparison, outcomes, and study) strategy in subjects with dental stress and anxiety (participants) treated with music therapy (intervention) in comparison with patients without music therapy (control) and evaluating the response to treatment (outcomes).

**Results:**

A total of 154 results were obtained, with 14 studies finally selected. The risk of bias and the methodological quality were assessed using the Cochrane Risk of Bias Tool and the Jadad scale, respectively. A random-effects meta-analysis was used to quantify the results of the pooled studies, while a fixed-effects meta-analysis was used for studies in the pediatric population. The meta-analysis of pooled studies found statistical significance in the subgroups of anxiety and anxiety–stress (*p* = 0.03 and *p* = 0.05, respectively), with an overall effect in favor of the intervention group (*p* = 0.005). Meta-analysis of the studies in the pediatric population showed considerable statistical significance for the experimental group (*p* < 0.00001).

**Conclusion:**

Music therapy as a treatment for stress and anxiety, prior to dental treatment, proved to be effective in both children and adults although more well-designed randomized clinical studies are needed to validate its efficacy.

**Systematic review registration:**

INPLASY, identifier 202312000.

## 1 Introduction

Anxiety is considered an emotional state that precedes confrontation with a stimulus and is distinguished from fear in that fear is the emotional response to a perceived threat (1). Both fear and dental anxiety are used interchangeably in the scientific literature although they represent progressive degrees of the same psychological state (2).

Dental treatments often cause, in patients who have to receive them, states of fear and anxiety, inducing them to avoid dental treatment, which results in a poorer quality of life in relation to oral health (3, 4).

There is some agreement that fear of dental treatment affects approximately one in five individuals although some studies place the prevalence at one in three (5–7). It has been known by clinicians that patients in this state, in addition to prolonging or even interrupting treatments, increase their economic costs and, in general, hinder dental practice, resulting in great frustration among dental health professionals (8, 9).

Dental anxiety has been shown to be a stress factor and is associated with personal traits, such as fear of pain, generally induced by unpleasant experiences of dental treatment in childhood or incited by others who have undergone such unpleasant experiences, as well as fear of blood or dental instruments (9, 10). These situations produce in the patient, in addition to psychological alterations, physiological ones, such as tachycardia, arterial hypertension, hyperthermia, mydriasis, hyperglycemia, and elevated cortisol levels, among others, which are caused by the activation of the hypothalamic–pituitary–adrenal axis that can lead to the development of certain systemic pathologies (11, 12).

Music is an auditory stimulus that includes, in addition to the melody itself, harmony, form, rhythm, timbre, and style, and music therapy (MT) is the clinical and evidence-based use of music interventions to achieve individualized goals within a therapeutic relationship by a credentialed professional who has completed an approved MT program (13).

The use of MT dates back to antiquity: the Greek philosopher Pythagoras stated that music exerted a positive influence on both the body and the soul, harmonizing both structures. In fact, philosophers of the Western world, from Pythagoras himself, Plato, and Aristotle to contemporaries including Schopenhauer and Nietzsche, have emphasized the healing power of music both for mental and bodily ailments (14). After the Second World War, MT began to be used conventionally, with the aim of accelerating the recovery of injured combatants, although previously, at the end of the 19th century, the psychology of music began to be studied, especially in the laboratories created in Germany and the USA (15). A good part of the current scientific literature has proposed MT as a positive modulator of patients’ physiological responses to anxiety (16).

In general, in patients with high anxiety in the dental room, anxiolytic drugs and conscious sedation have been used; however, studies have indicated that patients prefer non-pharmacological interventions, mainly due to the low risks involved (17). Therefore, listening to music to control fear and anxiety during dental procedures is widely accepted by adult patients, parents of pediatric patients, and professionals.

For all these reasons, this meta-analytical and systematic quantitative study aimed to examine and assess in the scientific literature the usefulness of MT in the management of stress/anxiety suffered by certain patients before undergoing dental treatments.

## 2 Methods

### 2.1 Study design and registration

This study was performed in accordance with the PRISMA (Preferred Reporting Items for Systematic Reviews and Meta-Analyses) statement (18) and the guidelines of the Clinical Practice Guidelines (19) (**Supplementary Table S1**, PRISMA checklist) and was registered in INPLASY no. 202312000 (DOI: 10.37766/inplasy2023.12.0008).

### 2.2 Question of interest: PICOS format

The question of interest was posed according to the PICOS (population, intervention, comparison, outcomes, and study) format: Is MT a tool that reduces or suppresses stress and anxiety in patients who are going to receive dental treatments? Intervention studies in humans who suffer anxiety before dental treatment (P) and that compared conventional dental treatment together with MT (I), *versus* subjects who only received conventional treatment (C), were considered with the aim of examining the results obtained for dental stress and anxiety (O) in randomized clinical studies (S) (**Table 1**).

**Table 1:** PICOS (population, intervention, comparison, outcomes, and study) format.

Population	Patients with stress and anxiety
Intervention	Conventional dental treatment + MT
Comparisons	Conventional dental treatment
Outcomes	To observe the effects of MT on the stress levels (salivary cortisol) (ΔSL) and/or values of anxiety levels (Corah’s Dental Anxiety Scale and Corah’s Modified Anxiety Scale) (ΔAL)
Study design	Randomized controlled trials (RCTs)

MT, music therapy; SL, stress level; AL, anxiety level; Δ, values obtained with experimental treatment.

### 2.3 Study selection and inclusion criteria

**Table 2** shows the inclusion and exclusion criteria. We searched for English-language studies that included musical interventions along with conventional oral treatments, rather than only traditional oral treatments, to manage dental stress and anxiety. All included research studies were categorized as randomized controlled clinical trials. Studies in which the full text could not be accessed, retrospective studies, case reports, reviews, and preclinical studies were not included. In addition, overlapping data from two or more studies or samples were excluded. The criteria and methodological operations were evaluated and performed separately by several people.

**Table 2:** Inclusion and exclusion criteria.

Inclusion criteria
1. English language
2. Conventional dental treatment + music therapy (experimental) and conventional dental treatment (control)
3. Outcome indicators: stress levels (cortisol in saliva) and anxiety levels (ΔSL and ΔAL)
4. Randomized clinical trials (RCTs)
Exclusion criteria
1. Studies in which full text cannot be accessed
2. Studies on non-conventional treatments (treatment of oral mucosal lesions, oral cancer, and resective surgery, among others)
3. Non-relevant articles (clinical cases, reviews, conference abstracts, meta-analyses, and preclinical studies, among others)
4. Small sample size (less than 5 subjects)
5. Duplicate studies in the databases consulted

### 2.4 Literature search

A computerized search of the electronic databases PubMed (*via* Medline), Web of Science (WOS), Embase, and the Cochrane Library was carried out up to October 2023. The following terms were used to formulate the search strategies: Anxiety Disorders* OR Stress Disorders* OR Dental Anxiety*/therapy OR Dental Fear* OR Phobic Disorders OR Dental Phobic/therapy* OR Dental Fear* AND Music Therapy* AND Humans* AND Randomized Controlled Trial*. The electronic search was supplemented with a manual search; moreover, the gray literature and the bibliographic references of the included studies were examined in order to obtain as much bibliographic information as possible and to minimize publication bias.

### 2.5 Data extraction and management

Two reviewers (NL-V and AL-V) independently performed a systematic screening of the titles and abstracts of the previously selected English-language registries. From the studies that met the inclusion criteria, the full text was read. Data extraction and disagreements between reviewers were resolved by discussion or consultation with a third reviewer (BMS); however, inter-reviewer agreement was assessed using Cohen’s kappa index (*κ*) (20). Although Cohen’s kappa (*κ*) suffers from a number of inconsistencies long debated by clinicians, it is considered the standard technique for diagnostic agreement and is used in the vast majority of analyses, having had its efficacy proven over the past 60 years.

### 2.6 Evaluation of study quality

To assess the methodological quality of the RCTs included in the meta-analysis, the Oxford Quality Scoring System or the Jadad Scale (21) was used, which takes into account biases related to randomization, masking, and loss to follow-up. The score assigned to each study ranged from 1 to 6 points, with those that obtained 5 and 6 points being considered studies of rigorous methodology, those that obtained 3 and 4 points of medium quality, and those that obtained scores lower than 3 points of low quality.

### 2.7 Risk of bias

The risk of bias was independently assessed using the Cochrane Risk of Bias Tool (RoB2) from the Cochrane Handbook for Systematic Reviews of Interventions (22) by two assessors (NL-V and AL-V). Five domains of bias were assessed: randomization process, deviations from intended interventions, missing outcome data, outcome measurement, and selection of reported outcomes. Studies with a high risk of bias were given a rating of “high,” those considered at low risk were rated “low,” while those with uncertainty bias or lack of information about possible bias were considered “borderline.” The studies included in the meta-analysis were classified as having a low, high, or borderline risk of bias. Discrepancies in the evaluation of RoB2 were discussed by NL-V and AL-V to reach a consensus.

### 2.8 Analysis

Data to evaluate the efficacy of MT on dental stress and anxiety were analyzed using Review Manager software (RevMan software, version 5.4.1; The Cochrane Collaboration, Copenhagen, Denmark, 2020).

Different meta-analyses were performed for anxiety and stress in children and adults. All were based on the mean difference (MD) and standard deviation (SD) to estimate the effect size, with 95% confidence intervals (CIs) to assess adverse outcomes. A random effects model was selected taking into account the uncertainty in *I* ^2^, considering the scarcity of studies in some situations (children) and the methodological heterogeneity found in the included studies. Heterogeneity was considered unimportant with *I* ^2 = ^0%–30%, moderate with *I* ^2 = ^40%–50%, substantial with *I* ^2 = ^60%–75%, and considerable with *I* ^2^ ≥ 75%. The threshold for statistical significance was set at *p* < 0.05. Meta-analysis of adverse outcomes was not performed due to lack of data reporting.

A sensitivity analysis was performed excluding studies that could lead to heterogeneity of the results and successive meta-analyses, each time excluding one of the selected trials. If any of the results were strongly discordant with the rest, the study that contributed the greatest degree of heterogeneity was identified.

## 3 Results

### 3.1 Study selection

**Figure 1:**
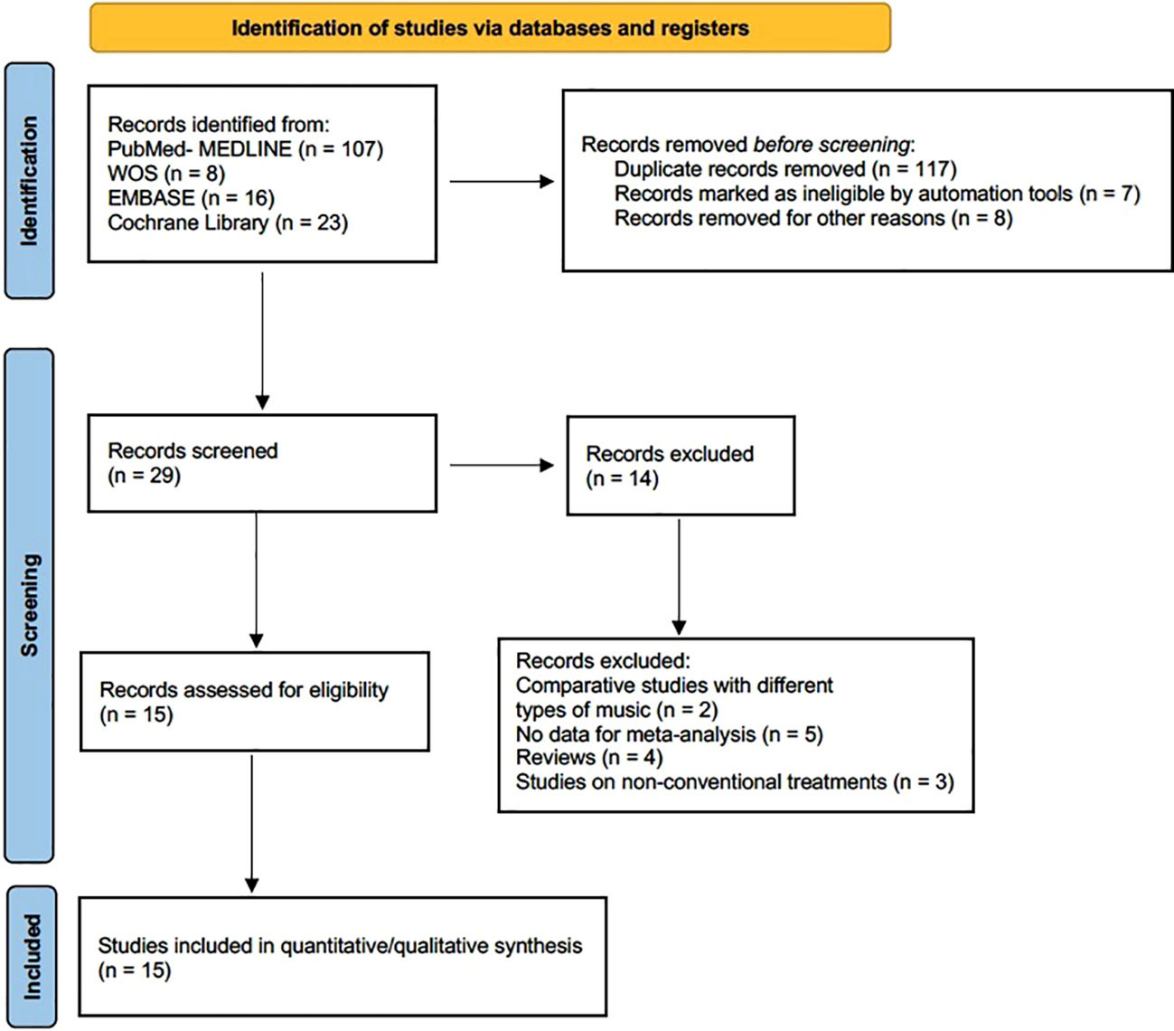
Flowchart of the study selection.

The four databases consulted yielded a total of 154 records. After eliminating 117 duplicates and 15 more as ineligible for automation tools and other reasons, 29 records were considered suitable. The full texts of these studies were obtained for further evaluation, after which 14 were excluded for cause. A total of 15 studies were included in the final analysis (**Figure 1**).

The discrepancy between the two reviewers (NL-V and AL-V) was 18%, resulting in high concordance (*κ* = 82%).

### 3.2 Study characteristics

The 15 studies included in the meta-analysis analyzed a sample of 1,402 subjects, of whom 308 were patients under 18 years of age. The largest samples were presented by the studies of Dixit and Jasani (23) (120 children), Sorribes de Ramón et al. (24) (275 adults), Kim et al. (25) (219 adults), and Janthasila and Keeratisiroj (26) (128 children).

Three studies evaluated a child population (26–28), while all the others included an adult population. The age range of the adult subjects included in the studies was between 18 and 57 years, while that of the child subjects was between 4 and 12 years.

Anxiety levels were assessed in all included studies. The study by Thoma et al. (29) assessed anxiety and stress, while those by Yamashita et al. (27) and Janthasila and Keeratisiroj (26) assessed anxiety and fear. The measurement scales used were Corah’s Dental Anxiety Scale (CDAS), Modified Dental Anxiety Scale (MDAS), North Carolina Behavior Rating Scale (NCBRS), The Dental Subscale of Children’s Fear Survey Schedule (CFSS-DS), State-Trait Anxiety Inventory (STAI), Hierarchical Anxiety Questionnaire (HAQ), Children’s Fear Survey Schedule (CFSS-DS), and Facial Image Scale (FIS). Salivary cortisol was measured by ELISA (30) and DRG (31). The music styles used varied between pop–folk and classical music.

The characteristics of the studies are presented in **Table 3**.

**Table 3:** Characteristics of the studies.

Study detail	Participant characteristics	Dental procedure	Music specification	Measurement tools	Outcomes	Funding
Lahmann et al. (32) Germany PRCS	**Sample size**: 90 **Sex**: 48 women **Age**: >18 years **Diagnosis**: Dental anxiety **Demographic**: NR **Others**: Subjects married or living with someone: 20 ± 71.4 MT, 26 ± 86.7 C Blue collar workers: 14 ± 50 MT, 11 ± 36.7 C Homemakers: 7 ± 25 MT, 9 ± 30 C	Restorative treatment of simple caries that were not in the advanced stage	NR	HAQ; STAI	MT significantly reduced dental anxiety; however, in very anxious subjects, it had no clinically relevant effect.	NR
Kim et al. (25) South Korea RCT	**Sample size**: 219 **Sex**: 99 women **Age**: >18 years **Diagnosis**: Dental anxiety **Demographic**: <40 years MT, <53 years C University education: 65 ± 61.3 MT, 73 ± 64.6 C Employed: 55 ± 51.9 MT, 51 ± 45.1 C **Others**: Reason for curative surgery: 51 ± 48.1 MT, 46 ± 40.7 C Reason for preventive surgery: 55 ± 51.9 MT, 67 ± 59.3 C	Mandibular third molar extraction	The patient selected his or her favorite songs from a list that included classical music, pop songs, folk songs, hymns, and Korean-style country songs.	CDAS	The music-treated group reported significantly less intraoperative anxiety than the control group (no music treatment)	NR
Mejía-Rubalcava et al. (33) Mexico REPS	**Sample size**: 34 **Sex**: 23 women **Age**: >18 years **Diagnosis**: Dental anxiety and stress **Demographic**: Housewife: 9 ± 52.9% Student: 3 ± 17.7% MT, 1 ± 5.8% C Employee: 5 ± 29.4% MT, 3 ± 17.7% C Education: Elementary, 2 ± 11.8%; high school, 9 ± 52.9% MT, 13 ± 76.4% C **Others**: Marital status: Single, 8 ± 47.0% MT, 2 ± 11.8% C Married/union: 7 ± 41.2% MT, 14 ± 82.3% C	Endodontic treatment Extraction	Music instrumentals soothing, calming, positive, relaxing daily “Essence No. 1” (iTunes)	MDAS; Concentration of cortisol in the saliva (Salimetrics, State College, PA, USA)	Significant differences were registered in the salivary cortisol concentration for the MT-treated group.	NR
Thoma et al. (29) Switzerland RCCT	**Sample size**: 92 **Sex**: 49 women **Age**: mean, 57 years **Diagnosis**: Dental anxiety and stress **Demographic**: Years of education: 13.56 ± 3.0 MT, 13.93 ± 3.01 C No. of appointments per year: 2.46 ± 1.07 MT, 2.33 ± 1.35 C **Others**: Trait dental anxiety: 34.6 ± 9.85 MT, 31.76 ± 9.34 C	Dental hygiene treatment	Relaxing music (“Miserere” by Allegri)	STAI; HAQ	The state anxiety levels in the music group decreased significantly after the intervention compared to the control group.	University of Zurich. Grant no. 56233208 (MVT)
Yamashita et al. (27) Thailand RCT	**Sample size**: 128 **Sex**: Women: 20 ± 60.6 MT, 13 ± 40.6 C **Age**: 20–40 years **Diagnosis**: Dental anxiety **Demographic**: Education: Grade 4, 13 ± 39.4 MT, 12 ± 37.5 C; Grade 5, 10 ± 30.3 MT, 8 ± 25.0 C; Grade 6, 10 ± 30.3 MT, 12 ± 37.5 C **Others**: Religion: Buddhism, 100%	Filling; extraction; scaling; application of fluoride sealant	NR	FIS; CFSS	Evaluation of the between-subjects effects revealed a significant effect of MT on dental anxiety and fear.	NF
Dixit and Jasani (23) India PGRCT	**Sample size**: 120 **Sex**: Women: 18 ± 45 MT, 14 ± 35 C **Age**: 4–6 years **Diagnosis**: Dental anxiety **Demographic**: NR **Others**: NR	Oral prophylaxis and fluoride treatment	Indian classical instrumental music (Raag Sohni played by Pandit Shiv Kumar Sharma on santoor)	FIS; NCBRS	Significant effects of exposure to MT on the reduction of dental anxiety in young children	NF
Aravena et al. (34) Chile PGRCT	**Sample size**: 42 **Sex**: 64.2% women **Age**: 15–40 years **Diagnosis**: Moderate level of dental anxiety and stress **Demographic**: NR **Others**: NI	Simple dental extraction	Two songs by Giorgio Costantini 2012 from the album “Universound”	Corah-MDAS; salivary cortisol levels (salivary cortisol: ELISA kit, Salimetrics Assays™, Pennsylvania, USA)	Significantly lower values of anxiety and salivary cortisol levels were observed in the music group compared to the control group.	NF
Kupeli and Gülnahar (35) Turkey ORCT	**Sample size**: 80 **Sex**: 44 women **Age**: 18–30 years **Diagnosis**: Moderate dental anxiety; high-level dental anxiety; extreme dental anxiety **Demographic**: NR **Others**: NR	Third molar extraction	Turkish music Classical music Rock music	CDAS	Anxiety levels decreased in all groups with different types of music; the postoperative CDAS values of the classical music group were significantly lower than those of the other groups.	NF
Gülnahar and Kupeli (36) Turkey PRCT	**Sample size**: 80 **Sex**: 47 women **Age**: 40–70 years **Diagnosis**: Extreme anxiety **Demographic**: NR **Others**: NR	Dental implant surgery	Classic Turkish music (Saba or Rast Tune Classical music (Vivaldi) Soft rock music	CDAS	All groups with music treatment presented a significant decrease in anxiety levels. It was observed that listening to music had a positive effect on dental anxiety regardless of the type of music. Postoperatively, Turkish music and classical music were much more effective in decreasing dental anxiety compared to soft rock music.	NF
Wazzan et al. (30) United Arab Emirates RCCT	**Sample size**: 46 **Sex**: Women 8 ± 17.4 **Age**: >18 years **Diagnosis**: Dental anxiety and stress **Demographic**: Education: Primary–high school, 25 ± 54.3; University, 18 ± 39.1 Visits to the dentists: First time, 21 ± 45.7; Previous visits, 25 ± 54.3 **Others**: Nationality: Arab, 20 ± 43.5; Non-Arab, 26 ± 56.5 Consumption of caffeine, alcohol, or nicotine: Yes, 16 ± 34.8; No, 30 ± 65.8	Dental treatment	Regular soft music	CDAS; cortisol in the saliva (salivary cortisol ELISA kit; Salimetrics Assays™, Pennsylvania, USA)	MT may help reduce salivary cortisol among patients in the intervention group who were exposed to slow-paced music compared to the control group treated without exposure to music; however, this reduction was not statistically significant.	NF
Kayaaltı-Yüksek and Yildirim (28) Turkey ORCT	**Sample size**: 60 **Sex**: 28 girls **Age**: 8–12 years **Diagnosis**: Dental anxiety **Demographic**: Plaque index: 0.82 ± 0.07 MT, 1.98 ± 0.08 C Gingival index: 0.77 ± 0.08 MT, 1 ± 0.07 C **Others**: NR	Oral hygiene training	First movement of Mozart’s Sonata for two pianos in D major, K. 448, during oral hygiene training (approximately 3 min)	CFSS	Listening to Mozart’s music before toothbrushing training had a significant effect on plaque removal in children with high dental anxiety.	NF
Esteban Pellicer et al. (37) Spain RCT	**Sample size**: 26 **Sex**: 13 women **Age**: 24–69 years **Diagnosis**: Dental anxiety **Demographic**: Anxiety level: 4.25 ± 3.92 MT, 2.63 ± 3.62 C **Others**: Pain level: 1.25 ± 1.75 MT, 0.75 ± 1.75 C	Dental implant surgery	Four Seasons (Vivaldi), Overture No. 3 (Bach), Adagio for Strings and Organ (T. Albinoni); Symphony N. 40, Sonata for 2 Pianos (Mozart) and Symphony N. 41 (Mozart)	Corah-MDAS; VAS	The incorporation of a musical flow in an individualized way into each patient in dental clinics is a useful non-pharmacological therapy to reduce anxiety in patients.	NF
Karapicak et al. (31) Turkey RCCT	**Sample size**: 70 **Sex**: 56 women **Age**: >18 years **Diagnosis**: Moderate dental anxiety and stress **Demographic**: Single: 27 ± 77.1 MT, 25 ± 71.4 C; Married: 8 ± 22.9 MT, 10 ± 28.6 C **Others**: High school: 10 ± 28.6 MT, 12 ± 34.3 C University: 25 ± 71.4 MT, 23 ± 65.7 C Duration of dental treatment (in minutes): 49.3 ± 9.2 MT, 46.0 ± 9.4 C	Restorative dental treatments	Each patient’s favorite music	MDAS; salivary cortisol levels (enzyme-linked immunosorbent assay kit; DRG, Germany)	The study was performed in a dental hospital during the COVID-19 pandemic, which could have impacted the anxiety levels during treatment. Moreover, since it is a cross-sectional study, it may not be reliable in determining the cause–effect relationship.	Karadeniz Technical University Scientific Research Project Coordination Unit
Sorribes De Ramón et al. (24) Spain RCCT	**Sample size**: 275 **Sex**: 56.4% women **Age**: 18–50 years **Diagnosis**: Anxiety **Demographic**: Laterality of third molar (%): Right, 43 ± 47.3 MT, 42 ± 46.2 C; Left, 485 ± 2.7 MT, 4953 ±.8 C **Others**: Winter classification (%): Vertical, 313 ± 4.1 MT, 384 ± 1.8 C; Mesioangular, 192 ± 0.9 MT, 171 ± 8.7 C; Horizontal, 323 ± 5.2 MT, 272 ± 9.7 C	Extraction of the third molar	“Introduction and Allegro for harp, flute, clarinet, and string quartet” by Ravel and “Aria” by J.S. Bach	STAI; VAS	In the patients in the MT group, a significant decrease in the scores for the levels of anxiety was observed.	NF
Janthasila and Keeratisiroj (26) Thailand RCCT	**Sample size**: 128 **Sex**: women: 206 ± 0.6 MT, 134 ± 0.6 C **Age**: 10–12 years **Diagnosis**: Dental anxiety **Demographic**: Education: Grade 4, 13 ± 39.4 MT, 12 ± 37.5) C; Grade 5, 10 ± 30.3 MT, 8 ± 25.0 C; Grade 6, 10 ± 30.3 MT, 12 ± 37.5 C **Others**: Religion: Buddhism, 32%	Filling Extraction Scaling Application of fluoride sealant	Thai pop instrumental folk songs	CFSS	The use of an MT program reduced anxiety and fear of dental services in school-aged children.	NF

PGRCT, parallel-group randomized clinical trial; PRCT, prospective randomized controlled trial; NR, none reported; NI, no interest; NF, not funded; MT, music treatment; C, control; HAQ, Hierarchical Anxiety Questionnaire; STAI, State-Trait Anxiety Inventory; CDAS, Corah’s Dental Anxiety Scale; Corah’s-MDAS, Modified Dental Anxiety Scale; RCT, randomized clinical trial; REPS, randomized experimental prospective study; RCCT, randomized controlled clinical trial; FIS, Facial Image Scale; CFSS, Children’s Fear Survey Schedule; NCBRS, North Carolina Behavior Rating Scale; ORCT, observational randomized controlled trial.

### 3.3 Methodological quality of the studies

Only one of the studies included in the meta-analysis (34) achieved a score on the Jadad scale compatible with a rigorous study (≥5 points). Medium quality (3 and 4 points) was achieved by 5 studies (24, 26, 28, 31, 37), while the rest of the studies were of poor quality (≤2 points) (**Table 4**).

**Table 4:** Scores of the included studies on the Jadad scale.

Study	Randomization	Adequate randomization method	Blinding	Double blinding	Appropriate blinding method	Dropouts	Total score
Lahmann et al. (32)	1	1	0	0	0	DNR	2^a^
Kim et al. (25)	1	1	0	0	0	DNR	2^a^
Mejía-Rubalcava et al. (33)	1	1	0	0	0	DNR	2^a^
Thoma et al. (29)	1	1	0	0	0	DNR	2^a^
Yamashita et al. (27)	1	1	0	0	0	DNR	2^a^
Dixit and Jasani (23)	1	1	0	0	0	DNR	2^a^
Aravena et al. (34)	1	1	1	1	1	DNR	5^b^
Kupeli and Gülnahar (35)	1	1	0	0	0	DNR	2^a^
Gülnahar and Kupeli (36)	1	1	0	0	0	DNR	2^a^
Wazzan et al. (30)	1	1	0	0	0	DNR	2^a^
Kayaaltı-Yüksek and Yildirim (28)	1	1	1	0	1	DNR	4^c^
Esteban Pellicer et al. (37)	1	1	1	0	1	DNR	4^c^
Karapicak et al. (31)	1	1	1	0	1	DNR	4^c^
Sorribes De Ramón et al. (24)	1	1	1	0	0	DNR	3^a^
Janthasila and Keeratisiroj (26)	1	1	0	0	0	1	3^a^

Each study was assigned a score of 0–6.

DNR, does not report.

a Poor quality (≤2 points).

b Rigorous study (≥5 points).

c Medium quality (3 and 4 points).

### 3.4 Risk of bias

**Figure 2:**
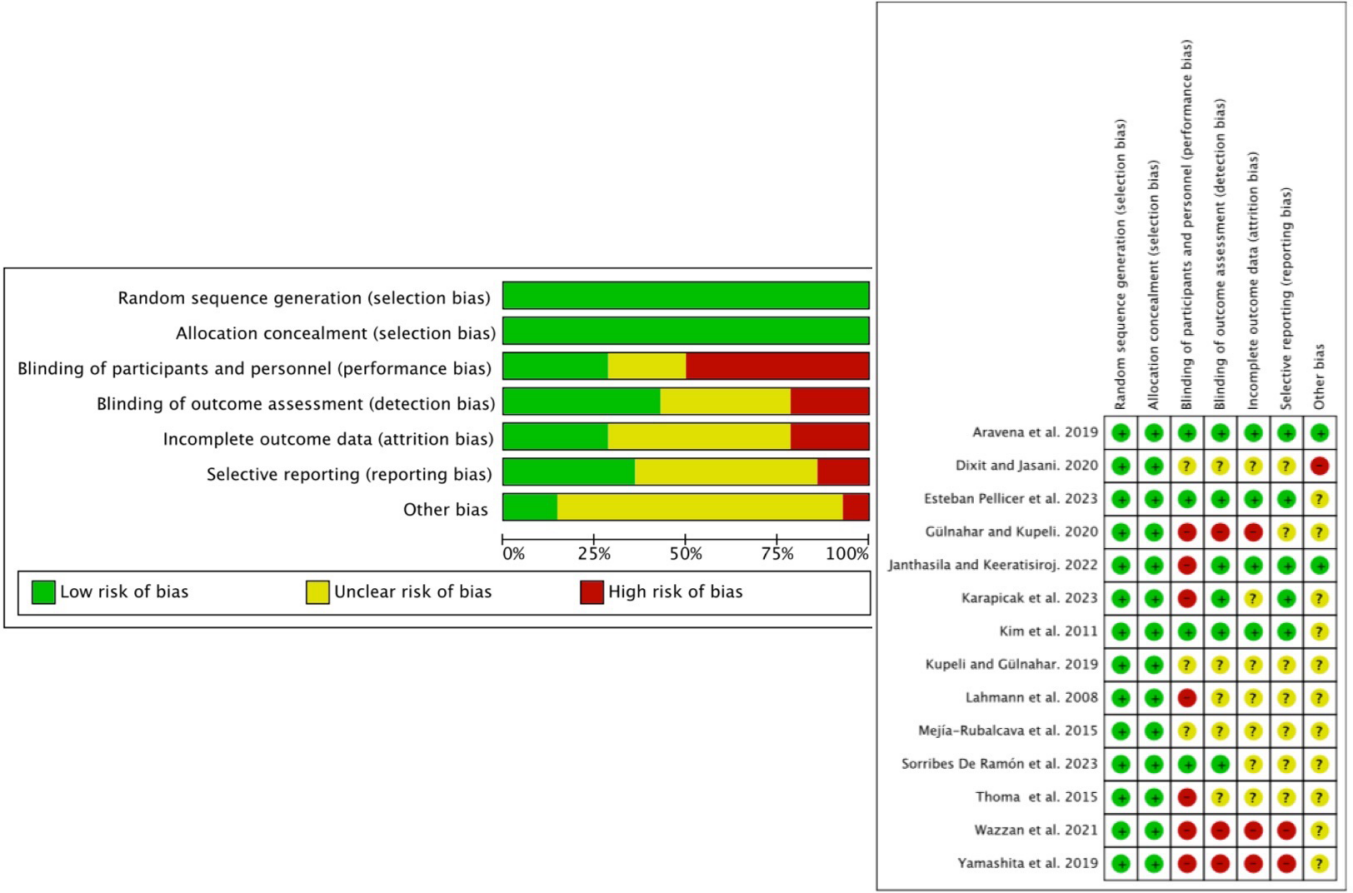
Risk of bias of the included studies.

Two reviewers (NL-V and AL-V) independently assessed the quality of the included studies, according to RoB2 (Cochrane Risk of Bias Tool) (22), based on 5 domains (i.e., randomization process, deviations from intended interventions, missing outcome data, outcome measurement, and selection of reported outcomes) and according to the Cochrane Handbook for Systematic Reviews of Interventions, outcome measurement, and selection of reported outcomes). According to the Cochrane Handbook for Systematic Reviews of Interventions, a “high” rating was given to studies considered with high risk of bias, “low” to those with low risk of bias, and “borderline” to those with uncertainty bias or lack of information on potential bias. Discrepancies between evaluators were settled by discussion and consensus. All included studies (23–37) addressed the domains “random sequence generation” (selection bias) and “allocation concealment” (selection bias), while the domain “blinding of participants and personnel” (performance bias) was met by only 4 studies (24, 25, 34, 37). The studies by Aravena et al. (34) and. Pellicer et al. (37) were the best rated. The study by Wazzan et al. (30) was the worst rated, together with the studies by Mejía Rubalcava et al. (33), Dixit and Jasani (23), and Kupeli and Gülnahar (35), which showed the greatest uncertainty (**Figure 2**). The risk of bias of the different subgroups is presented on the left of **Figure 2**.

### 3.5 Meta-analysis

For the meta-analysis, only 14 studies were included since the study by Kayaalti-Yüksek and Yildirim (28) evaluated the effectiveness of music in learning toothbrushing behavior in children with high and low levels of anxiety.

All of the studies in the adult population assessed dental anxiety. Stress was assessed in five studies (29–31, 33, 34), and only two studies (23, 26) assessed dental anxiety in the pediatric population.

**Figure 3:**
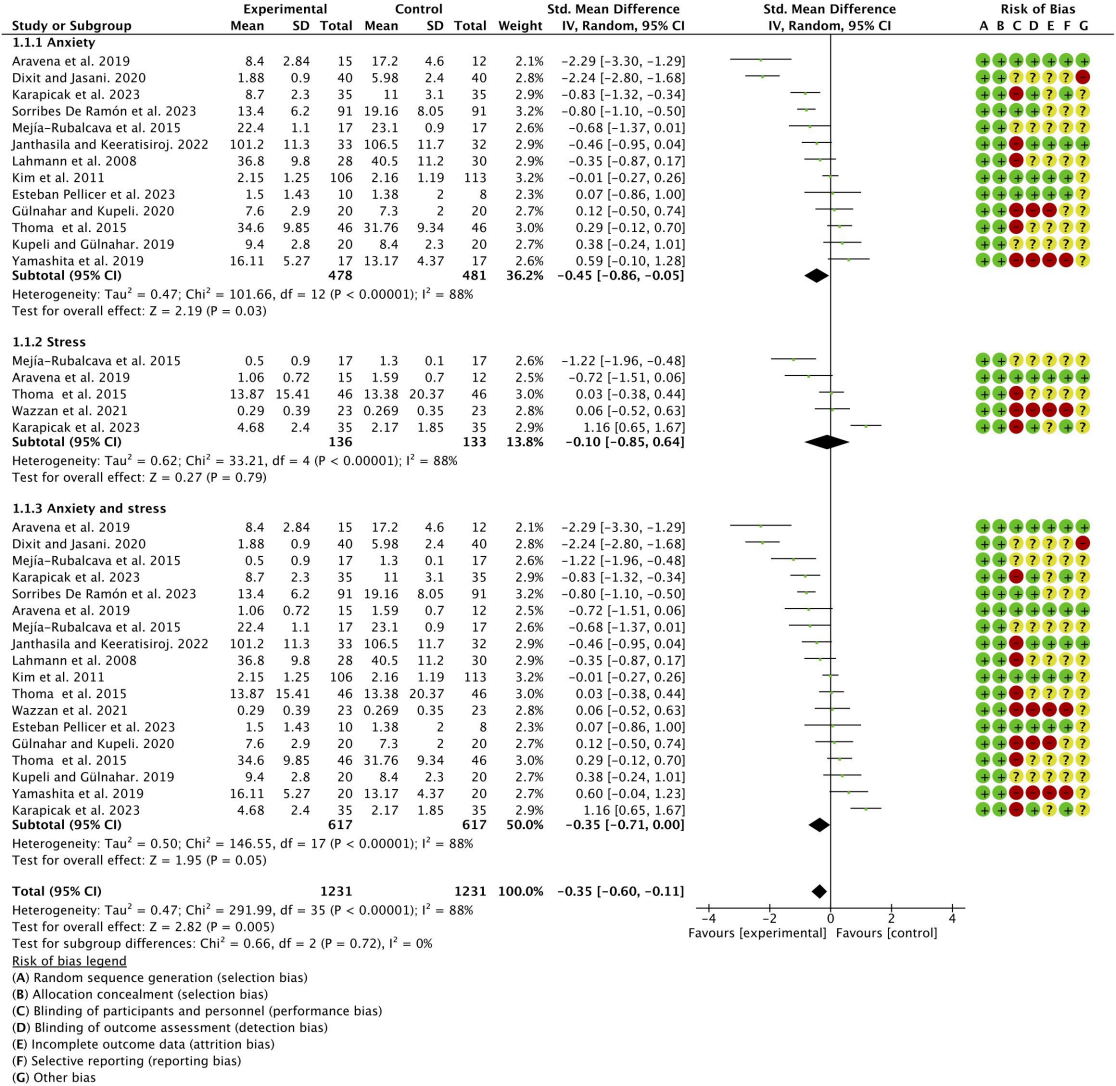
Meta-analysis (forest plot) by subgroups.

A meta-analysis by subgroups (anxiety, stress, and anxiety–stress) was performed. All subgroups presented considerable heterogeneity (*I* ^2^ > 75%); however, statistical significance was only found in the anxiety and anxiety–stress subgroups (*p* = 0.03 and *p* = 0.05, respectively), with an overall effect in favor of the experimental group (*p* = 0.005) (**Figure 3**).

**Figure 4:**
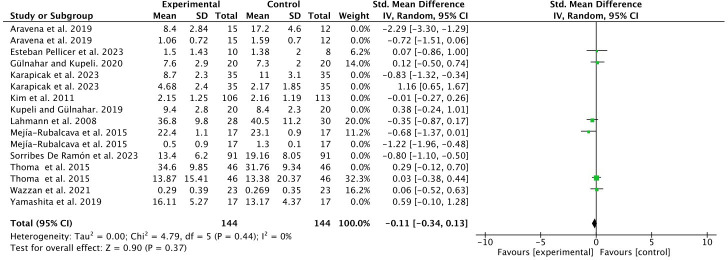
Sensitivity test in adults.

A sensitivity test was carried out in adults, discarding studies suspected of bias until a heterogeneity *I* ^2 ^= 0% was achieved (**Figure 4**).

**Figure 5:**
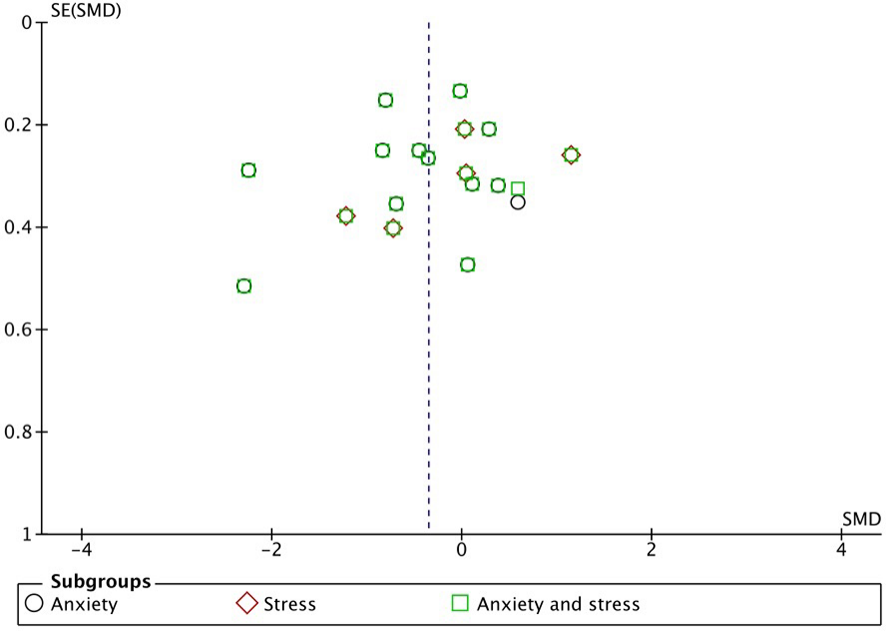
Forest plot in the child population.

Despite the paucity of studies on the effect of MT in the pediatric population (23, 26) and due to the considerable sample size (248 subjects), a meta-analysis was performed for this subgroup, resulting in zero heterogeneity (*I* ^2 ^= 0%) and considerable statistical significance in favor of the experimental group (*p* < 0.00001) (**Figure 5**).

A meta-analysis of adverse effects was also not performed due to the lack of data in the included studies.

### 3.6 Publication bias

**Figure 6:**
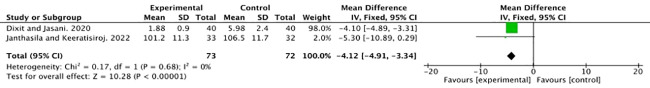
Funnel plot.

The graph in **Figure 6**, in which the abscissa axis (*X*) represents the observed results and the ordinate axis (*Y*) the standard error, shows low asymmetry and, therefore, low publication bias.

## 4 Discussion

Our meta-analysis found a positive association between the use of MT and the reduction of stress and anxiety in patients undergoing dental treatments.

Both routine visits to the dentist and the different dental treatments generally provoke different states of stress and anxiety in patients who have to receive them, depending, in general, on the complexity of the treatments. This leads to avoidance of dental treatments, with the consequent deterioration of health. Many of the patients who suffer from this type of alteration, especially high levels of dental anxiety, are treated with sedation, a technique that is costly and not without danger (38). For this reason, attempts have been made to develop effective and noninvasive psychological interventions to deal with this issue, with the aim of preventing patients from reducing dental care, which has important implications for public health (39).

Music has been used as a tool to improve well-being, reduce stress, and provide distraction to patients, which has been shown to be effective in reducing anxiety and improving mood in patients undergoing dental procedures and which is an affordable intervention (13).

The systematic review and meta-analysis presented here evaluated the effect of MT on stress and anxiety in patients undergoing dental treatments.

Several studies have investigated the effect of MT on stress and anxiety before and after the intervention (40–42). A Cochrane review of 20 studies suggested that listening to music may have a beneficial effect on anxiety in people awaiting surgery, in addition to highlighting that preoperative sedatives and anxiolytics often have negative side effects that lengthen patients’ recovery. Thus, offering musical interventions to patients was suggested as an alternative to these types of drugs (43). Another large-scale study indicated that listening to music produced greater benefits than midazolam in reducing preoperative anxiety (44). All these led to the increasing use of MT as a stress-reducing intervention in both medical and mental health settings (45).

The stress and anxiety that a good number of patients present before and during dental treatments justify the search for alternative, noninvasive, and inexpensive treatments without side effects that prevent the resistance of patients to receive dental treatments or diminish their quality, precisely because of this rejection. Based on these aspects, we conducted the present systematic review and meta-analysis, which had positive results and evaluated the effects of MT on stress and anxiety in patients undergoing dental treatments. Subjects in the experimental group with moderate, high, or extreme stress/anxiety states received MT and were compared to a control group that did not receive MT. Miyata et al. (46), in an RCT, estimated the effect of MT on preoperative anxiety by analyzing the heart rates in patients undergoing dental surgery under intravenous sedation and local anesthesia. The authors concluded that MT can alleviate anxiety by reducing sympathetic nervous activity; furthermore, they reported that MT would allow the treatment of stress on a continuous basis, i.e., from the patient’s arrival at the dental clinic for intravenous sedation until the completion of dental surgery, and concluded that taking into account the cost-effectiveness, the absence of adverse effects, the immediate effect, the safety in terms of no drug use, and the absence of concerns about recovery, MT would be instrumental in treating anxiety in dentistry. Other studies have also evaluated the role of MT in anxiety in patients undergoing dental extraction. A study conducted on a sample of 50 patients undergoing this intervention evaluated the anxiety levels in the experimental group treated with MT compared to the control group and found significant changes in both the hemodynamic levels and Corah’s-MDAS (47). Studies including large samples have evaluated the usefulness of different types of MT on anxiety levels, through the plasma noradrenaline level, in patients undergoing dental extraction, reporting benefits on anxiety in the experimental group treated with Islamic religious music (48).

In our meta-analysis, the anxiety scales and salivary cortisol values for dental anxiety and stress were evaluated as we considered these to be sufficient determinants for the diagnosis of these conditions. Cortisol is known as the stress hormone that is released in response to anxiety, while salivary cortisol is frequently used as a biomarker of psychological stress (49). We found statistical significance in the subgroups assessing anxiety and anxiety and stress combined (*p* = 0.03 and *p* = 0.05, respectively), with an overall effect in favor of the MT group. The meta-analysis of studies in the pediatric population, despite there being only two (23, 26) but with a large sample of patients, was particularly significant in favor of the experimental group (*p* < 0.00001).

Despite the results found on the effect of MT on stress and anxiety, we are aware of a series of limitations in this study. On the one hand, with respect to stress, only the salivary cortisol levels were considered, which we estimated to be more reliable than certain hemodynamic values, but which could be influenced by concomitant pathological situations (e.g., hypertension and paroxysmal tachycardia, among others). This was not clearly contemplated in many of the studies included in this meta-analysis, which could alter the results. Another aspect to consider would be the different types of scales used in the studies and their impact on the reported anxiety values, as well as the methods used to determine salivary cortisol. The differences between adults and children when assessing anxiety should also be mentioned.

A final aspect to take into consideration, which many patients report when receiving dental care, is the special odor that usually exists in dental clinics, generally due to the products used for the disinfection of surfaces and reusable instruments and the phenolic derivatives used as antiseptics in endodontic treatments, especially in teeth with necrotic pulp (50, 51). This aspect was covered by only one of the studies included in our meta-analysis (26).

Considering all these, our results should be taken with some caution.

## 5 Conclusions

The present systematic review and meta-analysis found that MT produced a beneficial effect on stress and anxiety control in patients receiving dental treatments; however, we believe that well-designed RCTs according to CONSORT standards are necessary and justifiable to corroborate this efficacy.

## Data availability statement

The original contributions presented in the study are included in the article/**Supplementary Material**. Further inquiries can be directed to the corresponding author.

## Author contributions

NL-V: Conceptualization, Data curation, Investigation, Writing – original draft, Writing – review & editing. AL-V: Writing – original draft, Writing – review & editing. BM: Data curation, Methodology, Writing – original draft, Writing – review & editing. JB: Supervision, Validation, Writing – original draft, Writing – review & editing.

## Supplementary material

Supplementary Table S1 Checklist.
