# Supplementary material for: Efficacy of music therapy on stress and anxiety prior to dental treatment: a systematic review and meta-analysis of randomized clinical trials
Source: Front Psychiatry. 2024 Feb 23;15:1352817. doi: 10.3389/fpsyt.2024.1352817 (PMC10920280; doi:10.3389/fpsyt.2024.1352817)
Supplement: Supplementary Table S1 — Checklist. [file Table_1.docx]

Table S1. Checklist

| **Section/topic** | **#** | **Checklist item** | | **Reported on page #** |
| --- | --- | --- | --- | --- |
| **TITLE: Efficacy of music therapy on stress and anxiety prior to dental treatment: A systematic review and meta-analysis of randomized clinical trials** | | | |  |
| Title | 1 | Efficacy of music therapy on stress and anxiety prior to dental treatment: A systematic review and meta-analysis of randomized clinical trials | | 1 |
| **ABSTRACT** | | | |  |
| Structured summary | 2 | Introduction- Stress and anxiety are emotional states that often accompany patients who have to receive dental treatments, leading them to postpone or avoid treatments with the consequent deterioration of their oral health and, consequently, their general condition. Music therapy (MT) has been shown to be an alternative treatment to other invasive and not without danger, such as anxiolytics or sedation. Our systematic review and meta-analysis evaluated the effect of music therapy on anxiety and stress prior to dental treatments. Methods- Studies published in PubMed (through Medline), Web of Science (WOS), Embase and Cochrane Library databases were consulted until October 2023. Inclusion criteria were established for intervention studies (RCTs), according to the PICOs strategy in subjects with dental stress and anxiety (participants), treated with music therapy (intervention) in comparison with patients without music therapy (control) and evaluating the response to treatment (outcomes). Results- A total of 154 results were obtained and finally, 14 studies were selected. The risk of bias was assessed using the Cochrane Risk of Bias Tool and the methodological quality using the Jadad scale. A random-effects meta-analysis was used to quantify the results of the pooled studies and a fixed-effects meta-analysis for the studies in the pediatric population. The meta-analysis of pooled studies found statistical significance in the subgroups of anxiety and anxiety-stress (p=0.03 and p=0.05 respectively), with an overall effect in favor of the intervention group (p=0.005). Meta-analysis of the studies in the pediatric population showed a considerable statistical significance for the experimental group (p<0.00001). Conclusions- Music therapy as a treatment for stress and anxiety, prior to dental treatment, proved to be effective in both children and adults, although more well-designed randomized clinical studies are needed to validate its efficacy. | | 1 |
| **INTRODUCTION** | | | |  |
| Rationale | 3 | Music is an auditory stimulus that includes, in addition to the melody itself, harmony, form, rhythm, timbre and style and music therapy is the clinical and evidence-based use of music interventions to achieve individualized goals within a therapeutic relationship by a credentialed professional who has completed an approved music therapy program.  The use of music MT dates back to antiquity; the Greek philosopher Pythagoras stated that music exerted a positive influence on both the body and the soul, harmonizing both structures. In fact, philosophers of the Western world, from Pythagoras himself, Plato and Aristotle, to contemporaries Schopenhauer and Nietzsche, have emphasized the healing power of music, both for mental and bodily ailments. After the Second World War, MT began to be used conventionally, with the aim of accelerating the recovery of injured combatants, although previously, at the end of the 19th century, the psychology of music began to be studied, especially in the laboratories created in Germany and the USA and a good part of the current scientific literature, proposes MT as a positive modulator of the patients' physiological responses to anxiety. | | 1,2 |
| Objectives | 4 | The objective of this quantitative meta-analytical and systematic approach was to examine the usefulness of MT in the management of stress/anxiety that certain patients suffer before undergoing dental treatment. | | 2 |
| **METHODS** | | | |  |
| Protocol and registration | 5 | *Study design and registration.* This study is presented in accordance with the PRISMA (Preferred Reporting Items for Systematic Reviews and Meta-Analyses) statement and the guidelines of the Clinical Practice Guidelines (Supplementary material, Table S1, PRISMA Checklist) and was registered in INPLASY number 202312000, DOI 10.37766/inplasy2023.12.0008. | | 3 |
| Eligibility criteria | 6 | PICOs: The question of interest was posed according to the PICOs format: Is MT a tool that reduces or suppresses stress and anxiety in patients who are going to receive dental treatments? Intervention studies in humans who suffer anxiety before dental treatment (P) and that compared conventional dental treatment together with MT (I), versus subjects who only received conventional treatment (C), were considered with the aim of observing the results obtained for dental stress and anxiety (O) in randomized clinical studies (s).  We searched for English-language studies that included musical interventions along with conventional oral treatments, rather than only traditional oral treatments, to treat dental stress and anxiety. All included research belonged to the category of randomized controlled clinical trials. Studies without access to the full text, retrospective studies, case reports, reviews or preclinical studies were not included. In addition, overlapping data from two or more studies or samples were eliminated. The criteria and methodological operations were performed separately, by several people. | | 3 |
| Information sources | 7 | PubMed/MEDLINE; WOS; EMBASE; The Cochrane Library | | 4 |
| Search | 8 | Present full electronic search strategy for at least one database, including any limits used, such that it could be repeated. | | 4 |
| Study selection | 9 | RCTs | | 4 |
| Data collection process | 10 | The titles and abstracts of the selected articles were collected and entered into an Excel spreadsheet, eliminating studies that did not refer to the research question. | | 4 |
| Data items | 11 | PICOs | | 4 |
| Risk of bias in individual studies | 12 | Two reviewers (NL-V and AL-V) independently assessed the quality of each RCT according to the Cochrane Risk of Bias Tool (RoB2) | | 5 |
| Summary measures | 13 | Difference in means. | | 5 |
| Synthesis of results | 14 | I^2^ for each meta-analysis. | | 5 |
| Section/topic | # | Checklist item | | Reported on page # |
| Risk of bias across studies | 15 | Funnel plot | | 15 |
| Additional analyses | 16 | ---------------- | |  |
| **RESULTS** | | | |  |
| Study selection | 17 | | The 15 studies included in the meta-analysis analyzed a sample of 1402 subjects, of whom 308 were patients under 18 years of age. The discrepancy between the two reviewers (NL-V, AL-V) was 18%, resulting in high concordance (κ=82%). | 6 |
| Study characteristics | 18 | | The largest samples were presented by the studies of Dixit and Jasani (120 children), Sorribes de Ramón et al (275 adults), Kim et al [24] (219 adults) and Janthasila and Keeratisiroj (128 children).  Three studies evaluated a child population and all the others, an adult population; the age range of the adult subjects included in the studies was between 18 and 57 years and that of the child subjects between 4 and 12 years. | 6 |
| Risk of bias within studies | 19 | | Two reviewers (NL-V and AL-V) independently analyzed the quality of the included studies, according to RoB2 (Cochrane Risk of Bias Tool), under 5 domains. All included studies addressed the domains "random sequence generation" (selection bias) and "allocation concealment" (selection bias); the domain "blinding of participants and personnel" (performance bias) was met by only 4 studies. The studies by Aravena et al and. Pellicer et al were the best rated and the study by Wazzan et al the worst rated, together with the studies by Mejía Rubalcava et al, Dixit and Jasani and Kupeli and Gülnahar, which showed the greatest uncertainty. | 13 |
| Results of individual studies | 20 | | ------------------------------- |  |
| Synthesis of results | 21 | | A meta-analysis by subgroups (anxiety; stress and anxiety-stress) was performed. All subgroups presented considerable heterogeneity (I^2^>75%), although statistical significance was only found in the anxiety and anxiety-stress subgroups (p=0.03 and p=0.05 respectively), with an overall effect in favor of the experimental group (p=0.005). Despite the paucity of studies on the effect of MT in the pediatric population and due to the considerable sample size (248 subjects), a meta-analysis was performed for this subgroup, resulting in zero heterogeneity (I^2^=0%) and considerable statistical significance in favor of the experimental group (p<0.00001) | 14 |
| Risk of bias across studies | 22 | | --------------------------------- |  |
| Additional analysis | 23 | | ---------------------------------- |  |
| **DISCUSSION** | | | | 10 |
| Summary of evidence | 24 | | ---------------------------------- |  |
| Limitations | 25 | | Despite the results found on the effect of MT on stress and anxiety, we are aware of a series of limitations that we will explain below: on the one hand, with respect to stress, we have only considered salivary cortisol levels, which we estimate to be more reliable than certain hemodynamic values, which could be influenced by concomitant pathological situations (hypertension, paroxysmal tachycardia, etc), not clearly contemplated in many of the studies included in this meta-analysis, which could alter the results; another aspect to consider would be the different types of scales used in the studies and their impact on the reported anxiety values, as well as the methods of detecting salivary cortisol; we should also mention the differences between adults and children when assessing anxiety.  A final aspect to take into consideration, that many patients report when receiving dental care, are the special odors that usually exist in dental clinics, generally due to products used for the disinfection of surfaces and reusable instruments and phenolic derivatives used as antiseptics in endodontic treatments, especially in teeth with necrotic pulp and this aspect is covered by only one of the studies included in our meta-analysis | 16 |
| Conclusions | 26 | | The present systematic review and meta-analysis found that MT produced a beneficial effect on stress and anxiety control in patients receiving dental treatments; however, we believe that well-designed RCTs according to CONSORT standards are necessary and justifiable to corroborate this efficacy. | 17 |
| **FUNDING** | | | |  |
| Funding | 27 | | No funding |  |
